# Clinical screening for GCK-MODY in 2,989 patients from the Brazilian Monogenic Diabetes Study Group (BRASMOD) and the Brazilian Type 1 Diabetes Study Group (BrazDiab1SG)

**DOI:** 10.20945/2359-4292-2023-0314

**Published:** 2024-07-30

**Authors:** Renata Peixoto-Barbosa, Luis Eduardo Calliari, Felipe Crispim, Regina S. Moisés, Sergio A. Dib, André F. Reis, Fernando M. A. Giuffrida

**Affiliations:** 1 Universidade Federal de São Paulo São Paulo SP Brasil Disciplina de Endocrinologia, Universidade Federal de São Paulo (Unifesp), São Paulo, SP, Brasil; 2 Departamento de Ciências da Vida Universidade do Estado da Bahia Salvador BA Brasil Departamento de Ciências da Vida, Universidade do Estado da Bahia (Uneb), Salvador, BA, Brasil; 3 Departamento de Pediatria Faculdade de Ciências Médicas Santa Casa de Misericórdia de São Paulo São Paulo SP Brasil Departamento de Pediatria, Faculdade de Ciências Médicas da Santa Casa de Misericórdia de São Paulo, São Paulo, SP, Brasil

**Keywords:** MODY, type 1 diabetes, glucokinase, diabetes mellitus, genetic techniques

## Abstract

**Objectives:**

To evaluate the accuracy of routinely available parameters in screening for GCK maturity-onset diabetes of the young (MODY), leveraging data from two large cohorts – one of patients with GCK-MODY and the other of patients with type 1 diabetes (T1D).

**Materials and methods:**

The study included 2,687 patients with T1D, 202 patients with clinical features of MODY but without associated genetic variants (NoVar), and 100 patients with GCK-MODY (GCK). Area under the receiver-operating characteristic curve (ROC-AUC) analyses were used to assess the performance of each parameter – both alone and incorporated into regression models – in discriminating between groups.

**Results:**

The best parameter discriminating between GCK-MODY and T1D was a multivariable model comprising glycated hemoglobin (HbA1c), fasting plasma glucose, age at diagnosis, hypertension, microvascular complications, previous diabetic ketoacidosis, and family history of diabetes. This model had a ROC-AUC value of 0.980 (95% confidence interval [CI] 0.974-0.985) and positive (PPV) and negative (NPV) predictive values of 43.74% and 100%, respectively. The best model discriminating between GCK and NoVar included HbA1c, age at diagnosis, hypertension, and triglycerides and had a ROC-AUC value of 0.850 (95% CI 0.783-0.916), PPV of 88.36%, and NPV of 97.7%; however, this model was not significantly different from the others. A novel GCK variant was also described in one individual with MODY (7-44192948-T-C, p.Ser54Gly), which showed evidence of pathogenicity on in silico prediction tools.

**Conclusions:**

This study identified a highly accurate (98%) composite model for differentiating GCK-MODY and T1D. This model may help clinicians select patients for genetic evaluation of monogenic diabetes, enabling them to implement correct treatment without overusing limited resources.

## INTRODUCTION

Maturity-onset diabetes of the young (MODY) encompasses various types of monogenic diabetes that account for 1%-5% of all diabetes cases (1). It is characterized by autosomal dominant inheritance, young-onset hyperglycemia, evidence of residual pancreatic function, and absence of beta-cell autoimmunity or insulin resistance (2). Variants associated with MODY have been reported in at least 11 genes to date (3,4).

Variants in glucokinase (*GCK*) and hepatocyte nuclear factor (*HNF1A/4A*) genes are the most common causes of MODY. Patients with GCK-MODY have mild, nonprogressive fasting hyperglycemia from birth, are typically asymptomatic, and their diagnosis is often incidental (5).

Despite its distinctive clinical features, GCK-MODY is often mistaken by clinicians for other common types of diabetes. On the one hand, patients with GCK-MODY share characteristics with those with type 2 diabetes, including insulin independence and absence of pancreatic autoimmunity. On the other hand, they resemble patients with type 1 diabetes (T1D) regarding age at diagnosis and lack of clinical insulin resistance. As a result, patients with GCK-MODY are often inadvertently treated with insulin or oral antidiabetic agents in routine clinical care, especially when diagnostic tools such as measurement of pancreatic islet antibodies and C-peptide levels are unavailable. Thus, MODY is frequently mislabeled, and it is estimated that more than 80% of patients potentially living with MODY remain without a correct molecular diagnosis (6).

In this context, two different precision medicine approaches to MODY should be considered: precision treatment and precision diagnosis (7). Precision treatment is a prototypical case of success in this field. Once a molecular diagnosis has been ascertained, individuals with GCK-MODY can have unnecessary treatments discontinued (8). Precision diagnosis, in contrast, still needs some fine-tuning. Despite the more widespread availability of molecular diagnosis, genetic testing is still expensive and, currently, universal testing is probably neither feasible nor cost-effective (9).

A widely used approach to determine the probability of MODY is a calculator tool proposed in a British study by Shields and cols. (10). Different groups, including those studying a Brazilian population, have sought to validate this tool (11,12). However, predictive models employing population-specific data are important for better screening individuals who must undergo molecular diagnosis. Therefore, this study aimed to evaluate the accuracy of routinely available clinical and laboratory parameters in discriminating GCK-MODY from T1D and clinical MODY without associated genetic variants (NoVar), leveraging data from two large Brazilian cohorts – one of patients with GCK-MODY and the other of patients with T1D.

## MATERIALS AND METHODS

### Patient recruitment

This study analyzed patients from the Brazilian Type 1 Diabetes Study Group (BrazDiab1SG) and the Brazilian Monogenic Diabetes Study Group (BRASMOD). The BrazDiab1SG is an observational, cross-sectional, multicenter study of 3,591 patients with T1D showing typical clinical presentation of overt diabetes (unequivocal clinical features of insulin deficiency or diabetic ketoacidosis at diagnosis) and continuous requirement of insulin since diagnosis. Pancreatic autoantibodies were not systematically measured for diagnosis since they are not widely available in participating study centers. The recruitment strategy of BrazDiab1SG has been detailed elsewhere (13). For the present study, we included 2,687 individuals from the BrazDiab1SG study in whom glycated hemoglobin (HbA1c) was assessed using methods certified by the National Glycohemoglobin Standardization Program (NGSP). The BRASMOD is a Brazilian national registry of patients with MODY with continuous and ongoing recruitment. Most patients included in the present study have been included in two previous studies (14,15), except for 12 cases, which are described here for the first time. All patients provided written consent (ethics committee protocol number CAAE 32784514.4.0000.5505). The methodology of the BRASMOD study has been described in the study’s original publications. Both BrazDiab1SG and BRASMOD were approved by local ethics committees at each participating institution.

The present study included 2,687 patients with T1D (T1D group), 202 patients with clinical features of MODY but without *GCK* or *HNF1A* variants by Sanger sequencing (NoVar group), and 100 patients with a genetic diagnosis of GCK-MODY (GCK group).

### Clinical and laboratory measurements

The following parameters, which were available for analysis, were obtained from the patients’ medical records: sex, age at diabetes diagnosis, age at recruitment, body mass index (BMI), BMI z-score (calculated using the LMS method) (16), systolic blood pressure, diastolic blood pressure, fasting plasma glucose, HbA1c, total cholesterol, high-density lipoprotein (HDL) cholesterol, low-density lipoprotein (LDL) cholesterol, triglycerides, fasting C-peptide, microalbuminuria, creatinine, presence of hypertension, previous episodes of diabetic ketoacidosis, and diabetes-related complications such as retinopathy, nephropathy, and neuropathy. Given the cross-sectional design of both datasets employed, the data were collected at unspecified points in the patients’ disease history.

### Genetic analyses

All individuals with GCK-MODY were diagnosed by Sanger sequencing as described in the original studies and were included only if they had either pathogenic (P) or likely pathogenic (LP) variants according to the American College of Medical Genetics and Genomics (ACMG) and Association for Molecular Pathology (AMP) (17), or variants of uncertain significance (VUS) according to the same criteria but with evidence of pathogenicity based on *in silico* prediction tools (18,19). Although the 202 patients in the NoVar group had previously only been tested for *GCK* and *HNF1A* variants, 24 of them took part in another study (20) where they underwent evaluation with a next-generation sequencing targeted panel of 11 genes (*HNF4A, GCK, HNF1A, PDX1, HNF1B, NEUROD1, CEL, INS, ABCC8, KCNJ11*, and *APPL1*). None of the patients had pathogenic or likely pathogenic variants, leading us to infer that these evaluated genes were not a major cause of diabetes in our dataset.

### Statistical analysis

Most variables showed a skewed distribution in at least one of the groups and we opted to employ nonparametric methods throughout the univariate analysis. Continuous variables were reported as median (interquartile range) and categorical variables were reported as counts (percentages). Since the only comparisons of interest were between GCK *versus* T1D and GCK *versus* NoVar, a global comparison among all three groups was not performed and we proceeded straight to pairwise comparisons, although all analyses have been corrected for three multiple comparisons. Continuous variables were analyzed by Dunn’s test and categorical variables were compared using pairwise Fisher’s test, both with Bonferroni correction. P values were capped at 1.0 whenever the corrected value was higher. All p values displayed were corrected, with p < 0.05 considered significant throughout the analyses. The logistic regression models were devised with either GCK *versus* T1D or GCK *versus* NoVar as binary outcomes, as described in the Supplementary Methods.

Receiver-operating characteristic (ROC) curve analysis was carried out first with each of the three most significant predictors from the univariate analysis and then with fitted values from the logistic models, employing the same binary outcomes as multivariable models. Areas under the ROC curve (ROC-AUCs) with 95% confidence intervals (CIs) were computed for each variable, and the optimal cutoff point was estimated using Youden’s J statistic (sensitivity + specificity - 1). Using only raw values from two-by-two tables to calculate predictive values would overestimate prevalence and thus falsely inflate calculations. Therefore, positive predictive values (PPVs) and negative predictive values (NPVs) were calculated using the equations described in the Supplementary Methods, employing prevalence (21). Since the prevalence of GCK-MODY is not entirely known, the following imputed prevalence values based on existing literature were employed: 0.5%, 1%, and 5% for GCK relative to T1D (22,23) and 10%, 40%, and 70% for GCK relative to NoVar (24).

Since all available GCK-MODY cases were included, a replication dataset was not available for external validation of cutoff and predictive values obtained in the analyses. Therefore, we proceeded with internal validation by bootstrapping. In brief, bootstrapping consists of drawing multiple simulated subsamples from a larger dataset and analyzing the distribution of the results from each simulation. The distribution of simulations usually approximates a normal distribution due to the central limit theorem and can, therefore, be used to compute CIs. This strategy was accomplished by the following simulation: drawing a random subsample of 50 individuals with GCK-MODY and 500 individuals with T1D, plotting a ROC curve for each parameter or multivariable model, and calculating ROC-AUC values. After 5,000 simulations, 95% bootstrap CIs were obtained utilizing the percentiles 50, 2.5, and 97.5 of the distribution of simulated ROC-AUC values. The same strategy was repeated to compare the GCK and NoVar groups, which included simulated samples of 50 and 100 individuals, respectively. All statistical analyses were performed using R, version 4.1.0 (R Foundation for Statistical Sciences, Vienna, Austria).

## RESULTS

### Univariate analysis

The study population’s clinical and laboratory data are shown in Table 1. The GCK group, compared with the T1D group, had a higher age at diagnosis (14 [9-27] years *versus* 10 [6-15] years, respectively, p = 2.98 x 10^-6^), higher age at recruitment (28 [10-41] years *versus* 19 [13-27] years, respectively, p = 0.0065), lower fasting plasma glucose level (118 [111-128] mg/dL *versus* 155 [100-248] mg/dL, p = 2.63 x 10^-5^), lower HbA1c level (6.3 [6.1-6.6]% *versus* 8.9 [7.7-10.7]%, p = 3.95 x 10^-32^), and lower prevalence rates of retinopathy (1% *versus* 10.1%, respectively, p = 0.0024), nephropathy (0% *versus* 11.6%, respectively, p = 3.56 x 10^-5^) and neuropathy (0% *versus* 5%, respectively, p = 0.043). A previous episode of diabetes ketoacidosis was not recorded in any of the patients with GCK-MODY but occurred in 161 (6.0%) of those with T1D (p = 0.0114).

**Table 1:** Clinical and laboratory features of the 2,989 study patients

	No variant	GCK	Type 1 diabetes	P value	

				GCK x No variant	GCK x Type 1 diabetes
n	202	100	2,687		
Female – n (%)	117 (58.2%)	57 (57%)	1,549 (57.6%)	1	1
Age at diagnosis (years)	25 [18-35]	14 [9-27]	10 [6-15]	9.52 x 10^-7^	2.98 x 10^-6^
Age at recruitment (years)	32 [21-49]	28 [10-41]	19 [13-27]	0.0035	0.0065
BMI (kg/m^2^)	24.7 [21.8-–28.5]	22.7 [19.4-25.6]	21.5 [18.8-24.2]	0.0037	0.2325
BMI z-score	0.8 [0.2-1.4]	0.3 [-0.3-1]	0.3 [-0.3-0.9]	0.0063	1
Systolic blood pressure (mmHg)	120 [120-130]	111 [110-120]	110 [100-120]	0.2547	0.0764
Diastolic blood pressure (mmHg)	80 [70-80]	78 [70-80]	70 [60-80]	1	0.0408
Hypertension – n (%)	45 (22.3%)	3 (3%)	345 (12.8%)	1.04 x 10^-5^	0.0051
FPG (mg/dL)	103 [89-149]	118 [111-128]	155 [100-248]	1	2.63 x 10^-5^
HbA1c (%)	7.0 [5.8-9.2]	6.3 [6.1-6.6]	8.9 [7.7-10.7]	3.18 x 10^-5^	3.95 x 10^-32^
Cholesterol (mg/dL)	178 [153-206]	171 [146-202]	165 [144-190]	1	0.3236
HDL cholesterol (mg/dL)	47 [39-58]	52 [43-59]	50 [43-60]	0.2103	1
LDL cholesterol (mg/dL)	100 [82-128]	97 [85-123]	96 [78-116]	1	0.6447
Triglycerides (mg/dL)	104 [72-170]	68 [50-104]	74 [54-107]	0.0001	1
C-peptide (ng/dL)	1.2 [0.74-1.58]	1.38 [1.02-1.8]	N/A	0.1004	N/A
AER (mg/g of creatinine)	5.8 [1-46.1]	6.4 [1.8-7.7]	10.4 [4.2-28.5]	0.5946	0.1300
Creatinine (mg/dL)	0.7 [0.6-0.8]	0.7 [0.6-0.8]	0.7 [0.6-0.9]	1	1
Diabetic ketoacidosis – n (%)	0 (0%)	0 (0%)	161 (6%)	1	0.0114
Retinopathy – n (%)	11 (5.4%)	1 (1%)	272 (10.1%)	0.3379	0.0024
Nephropathy – n (%)	9 (4.5%)	0 (0%)	311 (11.6%)	0.0971	3.56 x 10^-5^
Neuropathy – n (%)	3 (1.5%)	0 (0%)	135 (5%)	1	0.0430

Continuous variables are expressed as median [interquartile range], and categorical variables are expressed as absolute frequency (relative frequency). P values < 0.05 are significant. All p values have been adjusted for multiple comparisons. Abbreviations: AER, albumin excretion rate; BMI, body mass index, FPG, fasting plasma glucose; N/A, not available.

The GCK group, compared with the NoVar group, had lower age at diagnosis (14 [9-27] years *versus* 25 [18-35] years, respectively, p = 9.52 x 10^-7^), lower age at recruitment (28 [10-41] years *versus* 32 [21-49] years, respectively, p = 0.0035), lower BMI z-score (0.3 [-0.3-1] *versus* 0.8 [0.2-1.4], p = 0.0063), and lower HbA1c (6.3 [6.1-6.6]% *versus* 7.0 [5.8-9.2]%, p = 3.18 x10^-5^) and triglyceride (68 [50-104] mg/dL *versus* 104 [72-170] mg/dL, respectively, p = 0.0001) levels.

### Genetic analyses

Previously unpublished genetic data on 12 patients from the GCK group are described in this report. One novel variant was found in one of these patients: a T>C nucleotide substitution at position chr7:44,192,948, resulting in a p.Ser54Gly amino acid substitution. This variant was deemed a VUS according to the ACMG guidelines; however, it was considered possibly damaging according to *in silico* prediction by PolyPhen-2 (score 0.886), showed a REVEL score of 0.591 (denoting approximately 68% sensitivity, 92% specificity, and 9.0 likelihood ratio for being a true positive pathogenic variant), and was not found in gnomAD. The *GCK* variants are detailed in Table 2.

**Table 2:** Characteristics of GCK variants in 12 previously unpublished cases (amino acid substitutions and position on hg19 genome assembly, using the transcript ENST00000403799.3 as a reference)

Amino acid substitution	Position (hg19)	Exon	MAF (gnomAD)	ACMG classification	ACMG criteria	PolyPhen-2 score	REVEL Score	Number of affected individuals/families	Reference
p.Ser54Gly	7-44192948-T-C	2	0	VUS	PM2, PP2, PP4	0.886 (Possibly Damaging)	0.591	1/1	Unpublished
p.Ile130Val	7-44190650-T-C	4	0	LP	PM2, PM5, PP2, PP4	0.164 (Benign)	0.503	1/1	(43)
p.Arg186Ter	7-44189591-G-A	5	0	P	PVS1, PM2, PP4	N/A	N/A	1/1	(5)
p.Cys230Tyr	7-44187423-G-A	7	0	LP	PM2, PP1, PP2, PP3, PP4	0.997 (Probably Damaging)	0.966	2/1	(44)
p.Phe423Tyr	7-44184865-A-T	10	0	LP	PM2, PP1, PP2, PP3, PP4	0.993 (Probably Damaging)	0.801	5/2	(45)
p.Ala454Val	7-44184772-G-A	10	4.17E-06	LP	PM2, PM5, PP1, PP2, PP3, PP4	1.000 (Probably Damaging)	0.916	2/1	(43)

Abbreviations: ACMG: American College of Medical Genetics and Genomics; gnomAD, Genome Aggregation Database; LP, likely pathogenic; MAF, minor allele frequency; N/A, not applicable; P, pathogenic; PM, moderate pathogenic criterion; PolyPhen-2, Polymorphism Phenotyping v2; PP, supporting pathogenic criterion; PS, strong pathogenic criterion; PVS, very strong pathogenic criterion; REVEL, Rare Exome Variant Ensemble Learner; VUS, variant of uncertain significance.

### Multivariable models

Logistic regression models with GCK *versus* T1D as the binary outcome are described in Supplementary Table 1. Only HbA1c and age at diagnosis were independent predictors of GCK in all models. Model 2 had high p values due to categorical variables forced into the model, but demonstrated the best fit of all models. It had the lowest (268.65) Akaike Information Criterion (AIC; lower AIC values indicate a better model fit) and the highest R^2^ (0.71, *i.e.*, explaining 71% of the variability in the outcome).

Models with GCK *versus* NoVar as the binary outcome are shown in Supplementary Table 2. Age at diagnosis, HbA1c, and triglycerides remained as independent predictors in Models 4, 5, and 6 after adjustment. Model 5 showed the best fit (AIC of 128.29) and explained the highest variability in the outcome (R^2^ of 0.70).

### Receiver-operating characteristic curve analysis and bootstrap internal validation

**Figure 1:**
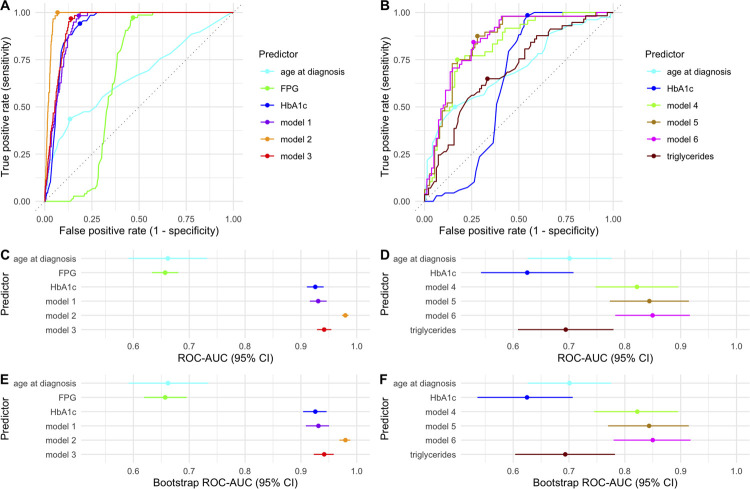
(A) Receiver-operating characteristic (ROC) curves of predictors significantly discriminating between GCK maturity-onset diabetes of the young (GCK-MODY) and type 1 diabetes and (B) between GCK-MODY and clinical maturity-onset diabetes of the young (MODY) without detected variants (NoVar). The dots plotted inside each curve in Panels A and B represent J points (maximum distance from the diagonal line). (C) Area under the ROC curve (ROC-AUC) and 95% confidence intervals (CIs) for each predictor shown in Panels A and (D) B. (E) Bootstrap ROC-AUC and 95% CIs for each predictor shown in Panels A and (F) B. The dots in Panels C to F represent ROC-AUC values and the horizontal colored bars represent their 95% CIs, enabling a visual comparison of significance among the predictors.

The ROC curves analyzing the performance of the predictors in discriminating between GCK-MODY and T1D are shown in Figure 1A. Model 2 (which included HbA1c, fasting plasma glucose, age at diagnosis, hypertension, presence of microvascular complications, history of diabetic ketoacidosis, and presence of a first-degree relative with diabetes) stood out as the best model. This was confirmed by the ROC-AUC 95% CIs depicted in the graph in Figure 1C, which shows that the ROC-AUC value for Model 2 (0.980, 95% CI 0.974-0.985) does not overlap with the ROC-ACU values of the other predictors, as the maximum upper boundary of their 95% CIs was 0.954. The 95% CIs obtained by bootstrapping, shown in Figure 1E, follow the same pattern seen in Figure 1C, confirming the findings.

Regarding models for discriminating GCK-MODY *versus* NoVar, none stood out as more accurate than the others. The ROC curves are depicted in Figure 1B. Despite Model 6 showing the highest ROC-AUC value (0.850, 95% CI 0.783-0.916), the 95% CIs of most predictors overlapped (Figure 1D). The same pattern is depicted in Figure 1F, which shows the 95% CIs obtained on bootstrapping.

### Predictive value analysis

Table 3 shows the cutoff values of predictors discriminating GCK-MODY *versus* T1D, with sensitivity and specificity estimated using the J statistic. The optimal cutoff value for Model 2, derived from the regression equation, was a fitted value of 0.043, which yielded a sensitivity of 100% and specificity of 93.23%. This model had an NPV of 100% for all imputed prevalences of GCK-MODY and PPVs that ranged from 6.91% to 43.74%. Since Model 2 had a significantly higher ROC-AUC value compared with the other predictors, it performed better in all settings.

**Table 3:** Estimated positive and negative predictive values of predictors discriminating GCK maturity-onset diabetes of the young (GCK-MODY) versus type 1 diabetes

				Imputed prevalence of GCK-MODY (relative to type 1 diabetes)					

				0.50%		1%		5%	
		
Predictor	Threshold	Sensitivity	Specificity	PPV	NPV	PPV	NPV	PPV	NPV
Age at diagnosis	19.5 years	43.59%	86.64%	1.61%	99.67%	3.19%	99.35%	14.65%	96.69%
FPG	149.5 mg/dL	97.30%	53.23%	1.03%	99.97%	2.06%	99.95%	9.87%	99.73%
HbA1c	7.35%	94.12%	81.35%	2.47%	99.96%	4.85%	99.93%	20.98%	99.62%
Model 1	0.026 (fitted value)	98.31%	81.93%	2.66%	99.99%	5.21%	99.98%	22.26%	99.89%
Model 2	0.043 (fitted value)	100.00%	93.23%	6.91%	100.00%	12.99%	100.00%	43.74%	100.00%
Model 3	0.032 (fitted value)	96.77%	86.14%	3.39%	99.98%	6.59%	99.96%	26.87%	99.80%

The thresholds, sensitivity, and specificity values (obtained using the J statistic) are shown for each predictor. The PPVs and NPVs are shown according to the imputed prevalence of GCK-MODY relative to type 1 diabetes. Model 1: HbA1c + FPG + age at diagnosis. Model 2: HbA1c + FPG + age at diagnosis + hypertension + history of diabetes in a first-degree relative + microvascular disease + diabetic ketoacidosis. Model 3: HbA1c + age at diagnosis + microvascular disease (backward elimination of Model 2). Abbreviations: FPG, fasting plasma glucose; HbA1c, glycated hemoglobin; NPV, negative predictive value; PPV, positive predictive value.

Table 4 shows analyses similar to those in Table 3, but with GCK-MODY *versus* NoVar as the outcome. Model 4 showed a high specificity (82.67%) at a cutoff value of 0.384 (fitted value), with a PPV ranging from 32.47% to 90.99%, depending on the imputed frequency of GCK-MODY. The highest sensitivity (98.53%) was for HbA1c alone at a cutoff value of 7.65%, which resulted in the highest NPVs of all predictors (92.98-99.64%). Model 6, in turn, showed the highest ROC-AUC value, with PPVs ranging from 26.54% to 88.36% and NPVs from 66.93% to 97.70%, depending on the imputed prevalence of GCK-MODY.

**Table 4:** Estimated positive and negative predictive values of predictors discriminating GCK maturity-onset diabetes of the young (MODY) versus clinical MODY without detected variants (NoVar)

				Imputed prevalence of GCK-MODY (relative to NoVar)					

				10%		40%		70%	
		
Predictor	Threshold	Sensitivity	Specificity	PPV	NPV	PPV	NPV	PPV	NPV
Age at diagnosis	14.5 years	50.00%	83.92%	25.67%	93.79%	67.45%	71.57%	87.88%	41.84%
HbA1c	7.65%	98.53%	45.45%	16.72%	99.64%	54.63%	97.89%	80.82%	92.98%
Model 4	0.478 (fitted value)	75.00%	82.67%	32.47%	96.75%	74.26%	83.22%	90.99%	58.63%
Model 5	0.347 (fitted value)	87.50%	72.00%	25.77%	98.11%	67.57%	89.63%	87.94%	71.17%
Model 6	0.384 (fitted value)	84.31%	74.07%	26.54%	97.70%	68.44%	87.63%	88.36%	66.93%
Triglycerides	84.5 mg/dL	64.91%	66.67%	17.79%	94.48%	56.49%	74.03%	81.96%	44.88%

The threshold, sensitivity, and specificity values (obtained using the J statistic) are shown for each predictor. The PPVs and NPVs are shown according to the imputed prevalence of GCK-MODY relative to NoVar. Model 4: HbA1c + age at diagnosis + BMI z-score + log(triglycerides). Model 5: HbA1c + age at diagnosis + BMI z-score + log(triglycerides) + hypertension. Model 6: HbA1c + age at diagnosis + log(triglycerides) + hypertension (backward elimination of Model 5). Abbreviations: BMI, body mass index; HbA1c, glycated hemoglobin; NPV, negative predictive value; PPV, positive predictive value.

## DISCUSSION

We presented herein a robust sample of 100 patients with confirmed variants in the *GCK* gene, 202 patients with clinical characteristics of MODY but no variants in either *GCK* or *HNF1A*, and 2,687 patients with T1D, obtained from data leveraged from two Brazilian multicenter cohorts.

Patients with GCK-MODY were older at diagnosis compared with those with T1D. However, age at diagnosis alone was not an accurate predictor in discriminating between diabetes subtypes. Patients with GCK-MODY have dysglycemia since birth and usually remain without a correct diagnosis for extended periods (25). Therefore, their higher age at diagnosis reflects this gap between identifying asymptomatic hyperglycemia and the correct determination of the etiology of diabetes. Gloyn and cols. have found that children and adolescents with hyperglycemia are more likely to have MODY than older individuals (>35 years); therefore, early screening of individuals younger than 25 years would facilitate the identification of MODY cases (26).

Probands with GCK-MODY may have a family history of clinical type 2 diabetes with no complications, or parents without known diabetes who have mildly increased fasting blood glucose levels (25). A family history of diabetes is an inclusion criterion in the BRASMOD study, and we considered it relevant to assess the potential role of this predictor in discriminating between GCK-MODY and T1D in the Brazilian population, given the high proportion (32.9%) of patients with T1D who had a first-degree relative with diabetes. A British study found that a family history of diabetes was the strongest predictor discriminating between T1D and MODY (27). Individuals with at least one parent affected with diabetes had approximately 23 times higher odds of having MODY than those with unaffected parents. Therefore, when GCK-MODY is suspected, assessing the parents’ fasting glucose and HbA1c levels can be a helpful strategy (28). Although most patients with GCK-MODY have inherited the variant from one of their parents, *de novo GCK* inactivating variants have been reported in the literature (29). Nevertheless, this rare finding has potentially little implication in the present setting.

Fasting glucose and HbA1c levels were lower in the GCK-MODY group compared with the T1D group. This finding is consistent with the predicted phenotype of patients with GCK-MODY, in whom fasting glucose level varies between 100-153 mg/dL and HbA1c level is usually between 5.6%-7.6%. Steele and cols. found that HbA1c effectively identified individuals with a *GCK* variant among those with clinical T1D (ROC-AUC value of 0.94) (30). In our model, HbA1c alone showed comparable discrimination between GCK-MODY and T1D at the threshold of 7.35%, with a ROC-AUC value of 0.93 (95% CI 0.91-0.94), 94.12% sensitivity, 81.35% specificity, and NPV greater than 99%.

The frequency of acute and chronic complications was extremely low among patients with GCK-MODY. In addition, there were no cases of diabetic ketoacidosis in this group, reflecting a preserved pancreatic function. This finding is consistent with other case series (31,32). Patients with a *GCK* variant have a low prevalence of microvascular and macrovascular complications despite being exposed to sustained lifelong mild hyperglycemia (15,33,34).

No differences in serum lipids were observed between the GCK-MODY and T1D groups. Fendler and cols. demonstrated that triglyceride levels were not helpful in discriminating between GCK-MODY and other types of diabetes. However, HDL cholesterol levels were lower in patients with a *GCK* variant than in those with T1D (35). A Chinese study found that patients with GCK-MODY had lower total cholesterol and LDL cholesterol levels than those with T1D (36). These divergent results could be explained by ethnic differences and issues related to sample sizes and therapies.

The most used model to select patients for genetic testing is the one proposed in 2012 by Shields and cols. The model evaluates patients diagnosed with diabetes before the age of 35 years. For MODY, compared with T1D, the most accurate discriminators in their study were lower HbA1c level, having a parent with diabetes, female sex, and older age at diagnosis (27). Except for sex, the findings of our study are similar to those of the study by Shields and cols. While the current UK standard for MODY testing relies on a PPV > 25%, other studies in non-Caucasian populations have found that higher cutoff values using the MODY probability calculator (above 62.5%) demonstrate high specificity and NPV (11,37). In a more recent Brazilian study, including a robust sample of patients with MODY, similar values (calculated probability above 60%) were found to be associated with good accuracy in selecting suspected MODY cases for molecular diagnosis (12).

Our study demonstrated that HbA1c was the best isolated parameter to discriminate between GCK-MODY and T1D. Furthermore, a model composed of HbA1c, fasting glucose, age at diagnosis, hypertension, presence of microvascular complications, history of diabetes in a first-degree relative, and a previous episode of diabetic ketoacidosis had excellent accuracy, resulting in a ROC-AUC value of 0.980 (95% CI 0.974-0.985).

In the analysis of predictors of GCK-MODY *versus* NoVar, no model emerged as superior to the others. However, the models provided a fair ability to distinguish GCK-MODY from NoVar. Although the ROC-AUC values were lower than those from the GCK-MODY *versus* T1D analysis, the higher imputed prevalence of GCK relative to NoVar resulted in higher predictive values. This strategy could still be useful in deciding among different sequencing strategies. While beyond the scope of the present study, other biomarkers could be incorporated into future studies to improve the discrimination between these diabetes subtypes (25).

The correct etiological diagnosis of GCK-MODY has important implications for the patients’ quality of life, treatment, and follow-up and for the allocation of health resources and genetic counseling. A US study found that 49% of patients with GCK-MODY were inadvertently treated and exposed to adverse events related to the use of oral antidiabetics. Although Latinos, African Americans, and Asians have a higher prevalence of diabetes in general, they accounted for only 20.5% of the probands screened in the study and 17.2% of those diagnosed with a *GCK* variant (38). This finding reinforces the need for studies including diverse populations.

The clinical phenotype of GCK-MODY is rather homogeneous compared to that of T1D, but many patients with GCK-MODY are misdiagnosed and inadequately treated. Despite advances in gene sequencing technologies, access to molecular testing is still not widely available or financially viable for most patients. A precision diagnosis approach to MODY should consider the prevalence and epidemiology of this type of diabetes, as well as its clinical features and diagnostic tests that should be robust, widely available, easy to perform, cost-effective, and approved by regulatory agencies (7). Therefore, incorporating additional biomarkers along with the clinical and laboratory parameters analyzed in the present study may enhance the selection process for molecular diagnosis, particularly when access is limited. This approach should remain practical and cost-effective.

The sample size and proportion of patients with T1D and GCK-MODY are some of the strengths of the present study. Another strength of the study is the development of potentially helpful screening strategies for diagnosing GCK-MODY using parameters widely available in clinical practice. This approach has enabled us to achieve high accuracy levels and, despite differences among populations, similar strategies could be useful in other groups. Making use of newer statistical tools, such as bootstrap simulation, allowed for internal replication, thus adding to the robustness of the data. However, some limitations of our study must be addressed. First, this was a cross-sectional study that employed random values of HbA1c. Thus, it was not possible to assess the implications of using the patients’ highest HbA1c value during longitudinal follow-up to rule out GCK-MODY. Likewise, other clinical and laboratory measurements were taken at different points in the patients’ disease history, making the cross-sectional design of our study unable to capture the longitudinal effect of therapy on such parameters. Second, only 13.2% of the evaluated patients reached the 7% target of HbA1c level in the BrazDiab1SG study. Although higher HbA1c values in our patients with T1D could potentially overestimate the role of HbA1c in discriminating between GCK-MODY and T1D, other large studies have shown similar HbA1c levels (39,40). Therefore, the difference between both groups regarding glycemic control could be seen as reflecting real-world data. Third, our sample was heterogeneous, since MODY patients were recruited using different inclusion criteria in the original studies. However, given the peculiar phenotype of GCK-MODY, we believe the large sample of 100 individuals compensated for any potential clinical heterogeneity. Fourth, the laboratory measurements were performed at different facilities. Still, no batch effect has been observed in MODY and T1D multicenter trials (data not shown). Fifth, we cannot exclude the possibility of MODY in the T1D group, as the inclusion criteria were based solely on clinical parameters, genetic testing was not carried out on all patients, and the diagnosis was not confirmed by pancreatic autoantibodies. However, other studies have also used clinical criteria for diagnosing T1D, reinforcing that, in many centers, the evaluation of pancreatic autoantibodies is not yet readily available on a large scale in clinical practice (39,41,42). Sixth, our study did not systematically evaluate other forms of MODY through molecular study. However, as described in the Methods section, around 12% of patients in the NoVar group were tested for 10 genes in addition to *GCK*, and none of them had pathogenic or likely pathogenic variants. Thus, we believe that even the possibility of misclassified patients would not have critically impacted our final model. Last, although we performed internal validation by bootstrapping, all our available GCK-MODY data were analyzed in the study and we were unable to conduct external validation.

In conclusion, our study demonstrated the feasibility of a highly accurate (98%) composite model in differentiating between GCK-MODY and T1D. This model may help clinicians select more appropriately those patients who should undergo genetic evaluation for monogenic diabetes, enabling correct treatment without overusing limited resources. However, an ideal model still needs to be established. There seem to be differences between populations in Brazilian and European studies regarding the accuracy of clinical criteria in GCK-MODY screening. Therefore, developing appropriate models for each population is paramount to optimize the selection of patients for genetic testing, enabling an accurate diagnosis of diabetes.

## SUPPLEMENTARY MATERIALS

Brazilian Monogenic Diabetes Study Group (BRASMOD) full member roster Brazilian Type 1 Diabetes Study Group (BrazDiab1SG) full investigator list Supplementary Methods


