# Supplementary material for: Clinical screening for GCK-MODY in 2,989 patients from the Brazilian Monogenic Diabetes Study Group (BRASMOD) and the Brazilian Type 1 Diabetes Study Group (BrazDiab1SG)
Source: Arch Endocrinol Metab. 2024 Jul 30;68:e230314. doi: 10.20945/2359-4292-2023-0314 (PMC11326741; doi:10.20945/2359-4292-2023-0314)
Supplement: Supplementary file 1 [file 2359-4292-aem-68-e230314-suppl01.pdf]

SUPPLEMENTARY MATERIALS

Brazilian Monogenic Diabetes Study Group (BRASMOD) full member roster
Brazilian Type 1 Diabetes Study Group (BrazDiab1SG) full investigator list
Supplementary Methods
Supplementary Results

- **Supplementary Table 1**
- **Supplementary Table 2**
- **Equations for MODY probability calculation derived from multivariable models**

Brazilian Monogenic Diabetes Study Group (BRASMOD) full member roster (group coordinators in bold): Universidade do Estado da Bahia, Salvador, BA, Brasil/Centro de Diabetes e Endocrinologia do Estado da Bahia (Cedeba), Salvador, BA, Brasil/Escola Paulista de Medicina, Universidade Federal de São Paulo, São Paulo, SP, Brasil: **Fernando M.A. Giuffrida.** Escola Paulista de Medicina, Universidade Federal de São Paulo, São Paulo, SP, Brazil: **André F. Reis**; Regina C. S. Moises; Renata P. Dotto; Sergio A. Dib; Magnus R. Dias-da-Silva; Antônio R. Chacra; Ilda S. Kunii; Carolina Soares Viana de Oliveira; Gilberto K. Furuzawa, Luciana Franco, João Roberto de Sá; Faculdade de Medicina da Universidade de São Paulo, São Paulo, SP, Brasil: Thais Della Manna; Monogenic Diabetes Group, Genetic Endocrinology Unit and Laboratory of Molecular & Cellular Endocrinology/LIM25, School of Medicine, University of São Paulo (USP), São Paulo, SP, Brasil: Lilian A. Caetano, Milena G. Teles; Faculdade de Ciências Médicas da Santa Casa de Misericórdia de São Paulo, São Paulo, SP, Brasil: Luis Eduardo Calliari; Universidade Federal do Rio Grande do Sul, Porto Alegre, RS, Brasil: Letícia S. Weinert, Sandra P. Silveiro; Unidade de Endocrinologia Pediátrica, Hospital Universitário Prof. Edgard Santos, Faculdade de Medicina, Universidade Federal da Bahia, Salvador, BA, Brasil: Renata Andrade Lima, Crésio Alves; Centro de Diabetes e Endocrinologia do Estado da Bahia (CEDEBA)/Escola Bahiana de Medicina e Saúde Pública, Salvador, BA, Brasil: Caroline Bulcão.

Brazilian Type 1 Diabetes Study Group (BrazDiab1SG) steering committee: Sergio A. Dib; Marilia B. Gomes; Carlos A. Negrato. **BrazDiab1SG investigator full list, with respective affiliations (principal investigators are marked with asterisks and program coordinators are underlined):** Universidade do Estado Rio de Janeiro: Roberta Cobas*, Alessandra Matheus, Lucianne Tannus; Universidade Federal Rio de Janeiro: Lenita Zajdenverg*, Melanie Rodacki; Hospital Geral de Bonsucesso: Neuza Braga Campos de Araujo*, Marilena de Menezes Cordeiro; Hospital Universitário Clementino Fraga Filho – IPPMG: Dr. Jorge Luiz Luescher*; Renata Szundy Berardo; Serviço de Diabetes da Disciplina de Endocrinologia e Metabologia do Hospital das Clínicas da Universidade de São Paulo: Marcia Nery*; Catarina Cani; Maria do Carmo Arruda Marques; Unidade de Endocrinologia Pediátrica da Santa Casa de Misericórdia de São Paulo: Luiz Eduardo Calliari*, Renata Maria de Noronha; Instituto da Criança do Hospital das Clínicas da Universidade de São Paulo: Thais Della Manna*, Roberta Salvodelli, Fernanda Garcia Penha; Hospital das Clínicas da Faculdade de Medicina de Ribeirão Preto – USP: Milton Cesar Foss*, Maria Cristina Foss-Freitas; Ambulatório da Faculdade Estadual de Medicina de São José do Rio Preto: Antônio Carlos Pires*, Fernando Cesar Robles; Associação de Diabéticos de Bauru: Carlos Antônio Negrato*, Maria de Fátima Soares Guedes; Centro de Diabetes da Escola Paulista de Medicina: Sergio Atala Dib*, Patricia Dualib; Clínica de Endocrinologia da Santa Casa de Belo Horizonte Setor Diabetes Tipo 1: Saulo Cavalcanti da Silva*, Janice Sepulveda; Ambulatório Multiprofissional de Atendimento a Diabetes do Hospital de Clínicas da Universidade Estadual de Londrina: Henriqueta Guidio de Almeida*, Emerson Sampaio; Hospital de Clínicas da Universidade Federal do Paraná: Rosangela Roginski Rea*, Ana Cristina Ravazzani de Almeida Faria; Instituto da Criança com Diabetes do Rio Grande Sul: Balduino Tschiedel*, Suzana Lavigne, Gustavo Adolfo Cardozo; Hospital de Clínicas de Porto Alegre: Mirela Azevedo*, Luis Henrique Canani, Alessandra Teixeira Zucatti; Hospital Universitário de Santa Catarina: Marisa Helena Cesar Coral*, Daniela Aline Pereira; Instituto de Diabetes-Endocrinologia de Joinville: Luiz Antônio de Araújo*; Hospital Regional de Taguatinga, Brasília: Hermelinda Cordeiro Pedrosa*,

Copyright© AE&M all rights reserved.

Monica Tolentino; Flaviene Alves Prado; Hospital Geral de Goiânia Dr. Alberto Rassi: Nelson Rassi*, Leticia Bretones de Araujo; Centro de Diabetes e Endocrinologia do Estado da Bahia: Reine Marie Chaves Fonseca*; Alexis Dourado Guedes, Odelisa Silva de Mattos; Universidade Federal do Maranhão: Manuel Faria*, Rossana Azulay; Centro Integrado de Diabetes e Hipertensão do Ceará: Adriana Costa e Forti*, Maria Cristina Façanha; Universidade Federal do Ceará: Renan Montenegro Junior*, Ana Paula Montenegro; Universidade Federal de Sergipe: Naira Horta Melo*, Karla Freire Rezende; Hospital Universitário Alcides Carneiro: Alberto Ramos*; Hospital Universitário João de Barros Barreto, Pará: Joao Felício Soares*, Flavia Marques Santos; Hospital Universitário Getúlio Vargas, Hospital Adriano Jorge: Deborah Laredo Jezini*.

SUPPLEMENTARY METHODS

The logistic regression models were devised using either GCK-MODY *versus* T1D or GCK-MODY *versus* NoVar as the binary outcome. Significant variables from univariate analyses were entered as predictors in logistic models in three ways: (A) all significant continuous predictors, (B) all significant predictors (both continuous and categorical), and (C) backward elimination (progressive exclusion of predictors with the highest p value until only significant predictors remained). The models were named Models 1, 2, and 3 (GCK *versus* T1D as the binary outcome) and 4, 5, and 6 (GCK *versus* NoVar as the binary outcome). Fitted values of regression models were computed for each patient. The Akaike Information Criterion (AIC) was calculated as described below, and R^2 was computed for each model.

Equation 1. Probability calculation employing the logit obtained from logistic regression:

$$p = \frac{e^{logit}}{1+e^{logit}}$$

Equation 2. Akaike Information Criterion (AIC) calculation:

$$AIC = -2 * log\ (likelihood) + 2 * (number\ of\ predictors)$$

Equation 3. Positive predictive value (PPV) calculation employing sensitivity (Se), specificity (Sp), and prevalence (prev):

$$PPV = \frac{(Se * prev)}{(Se * prev) + [(1 - Sp) * (1 - prev)]}$$

Equation 4. Negative predictive value (NPV) calculation employing sensitivity (Se), specificity (Sp), and prevalence (prev):

$$NPV = \frac{[Sp * (1 - prev)]}{\{[Sp * (1 - prev)] + [(1 - Se) * prev]\}}$$

Copyright© AE&M all rights reserved.

Supplementary table 1. Logistic regression models with GCK-MODY *versus* T1D as the binary outcome

Models	Beta	p	AIC	R^2
**Model 1**				
HbA1c	-1.085	1.70E-17	397.50	0.55
FPG	-0.004	0.1421		
Age at diagnosis	0.075	1.27E-07		
**Model 2**				
HbA1c	-1.093	8.2E-11	268.65	0.71
FPG	-0.006	0.0810		
Age at diagnosis	0.072	1.95E-05		
Microvascular complications	-2.251	0.0342		
Hypertension	-1.545	0.0589		
Diabetic ketoacidosis	-17.325	0.9952		
First-degree relative with DM	-19.598	0.9839		
**Model 3**				
HbA1c	-1.141	3.10E-19	395.57	0.55
Microvascular complications	-2.834	0.0057		
Age at diagnosis	0.084	5.63E-09		

Abbreviations: AIC, Akaike Information Criterion; DM, diabetes mellitus; HbA1c, glycated hemoglobin; FPG, fasting plasma glucose.

Supplementary table 2. Logistic regression models with GCK-MODY *versus* no variant (NoVar) as the binary outcome

Models	Estimate	p	AIC	R^2
**Model 4**				
Age at diagnosis	-0.039	0.0339	133.21	0.68
BMI z-score	-0.254	0.1620		
HbA1c	-0.366	0.0066		
Log(triglycerides)	-1.221	0.0077		
**Model 5**				
Age at diagnosis	-0.037	0.0451	128.29	0.70
BMI z-score	-0.205	0.2999		
HbA1c	-0.265	0.0531		
Log(triglycerides)	-1.15	0.0171		
Hypertension	-1.84	0.0228		
**Model 6**				
Age at diagnosis	-0.045	0.0119	133.99	0.68
HbA1c	-0.296	0.0312		
Log(triglycerides)	-1.191	0.0083		
Hypertension	-1.875	0.0193		

Abbreviations: AIC, Akaike Information Criterion; BMI, body mass index; FPG, fasting plasma glucose; HbA1c, glycated hemoglobin.

Copyright© AE&M all rights reserved.

Equations for MODY probability calculation derived from multivariable models. Continuous predictors should be entered as numeric values and categorical predictors should be entered as either 0 (absent) or 1 (present) in the equations. Results obtained from the equations below are logits or log-odds. Fitted probabilities should be calculated with the formula provided above, in Supplementary Methods. Abbreviations: DKA, diabetes ketoacidosis; HbA1c, glycated hemoglobin.

$$\boldsymbol{logit}_{\boldsymbol{GCK\ x\ Type\ 1\ diabetes}}$$
$$= -13.904 - 1.093 * HbA1c - 0.006 * FPG + 0.072 * Age\ at\ diagnosis$$
$$- 2.251 * Microvascular\ complications - 1.545 * Hypertension$$
$$- 17.325 * DKA + 19.598 * First - degree\ relative\ with\ diabetes$$

$$\boldsymbol{logit}_{\boldsymbol{GCK\ x\ NoVar}}$$
$$= 8.371 - 0.045 * Age\ at\ diagnosis\ - 0.296 * HbA1c - 1.191$$
$$* log(triglycerides) - 1.875 * Hypertension$$

This is an open-access article distributed under the terms of the Creative Commons Attribution License

Copyright© AE&M all rights reserved.
